# Lack of Association between *Toxocara* Exposure and Suicide Attempts in Psychiatric Patients

**DOI:** 10.1155/2015/608604

**Published:** 2015-09-21

**Authors:** Cosme Alvarado-Esquivel, Jesús Hernández-Tinoco, Luis Francisco Sánchez-Anguiano

**Affiliations:** ^1^Biomedical Research Laboratory, Faculty of Medicine and Nutrition, Juárez University of Durango State, 34000 Durango, DGO, Mexico; ^2^Institute for Scientific Research “Dr. Roberto Rivera Damm”, Juárez University of Durango State, 34000 Durango, DGO, Mexico

## Abstract

Infection with *Toxocara* may affect the central nervous system. A high seroprevalence of *Toxocara* infection has been reported in psychiatric patients. To the best of our knowledge, there is no previous report about an association of *Toxocara* infection with suicide attempts. Therefore, we sought to determine whether *Toxocara* exposure is associated with suicide attempts in psychiatric patients. We studied 282 psychiatric outpatients (156 with suicide attempts and 126 without suicide attempts). Sera of patients were analyzed for the presence of anti-*Toxocara* IgG antibodies by using a commercially available enzyme immunoassay. One of the 156 (0.6%) suicide attempters and 1 (0.8%) of the 126 controls were positive for anti-*Toxocara* IgG antibodies (OR = 0.80; 95% CI: 0.04–13.02; *P* = 1.00). *Toxocara* seropositivity was significantly higher (*P* = 0.01) in male patients with consumption of raw dried goat meat than male patients without this consumption. Results suggest that *Toxocara* exposure is not associated with suicide attempts in psychiatric outpatients in Durango City, Mexico. However, further studies with larger samples sizes to confirm our results should be conducted. Too few patients were seropositive to assess further associations of *Toxocara* exposure with sociodemographic, clinical, and behavioral characteristics of the psychiatric patients.

## 1. Introduction

The nematode parasite* Toxocara* causes infections in intestines of dogs and cats [1]. Local environment is contaminated with parasite eggs shed by infected cats and dogs [2, 3]. Humans acquire an infection with* Toxocara* by accidental consumption of infecting parasite eggs or larvae [4, 5]. Infection with* Toxocara* is one of the most widespread zoonotic parasitic infections [4] and causes a disease known as toxocariasis [4]. The burden of toxocariasis in North America is significant [6]; however, toxocariasis is recognized as a neglected zoonotic disease [6, 7]. The seroprevalence of* Toxocara* infection varies substantially among population groups, that is, 2%–5% in adults in urban areas, 14.2%–37% in rural areas, and 63.2%–92.8% in some tropical countries [8].* Toxocara* does not mature in the human intestines but instead migrates through tissues and organs of the body [1]. Dissemination of* Toxocara* may occur to muscles, eyes, liver, lungs, and central nervous system [7]. Infections with* Toxocara* are usually asymptomatic [4, 7]. However, some infections may lead to severe toxocariasis and death [9].* Toxocara* infection of the eye is a major cause of blindness [10]. Visceral toxocariasis is commonly associated with liver and pulmonary damage [6]. Less commonly, toxocariasis may manifest with pericardial effusion or myocarditis [6].* Toxocara* may invade the brains of humans [11]; however, neurotoxocariasis or cerebral toxocariasis remains a poorly understood phenomenon [1].* Toxocara* infection may lead to eosinophilic meningitis [6], meningoencephalitis, myelitis, cerebral vasculitis, optic neuritis [12], epilepsy [13], and multiple cerebral infarction [14]. In addition, toxocariasis has been associated with dementia [11, 15, 16] and mental confusion [17]. A high seroprevalence of* Toxocara* infection has been found in psychiatric patients [18–20]. In a study in Italy, researchers found a 13% seroprevalence of* Toxocara* infection in psychiatric patients [18]. In a recent study in Mexico, 4.7% of 128 psychiatric inpatients were seropositive for* Toxocara* infection [19], whereas, in a study in China, a 16.4% seroprevalence of* Toxocara* infection in psychiatric patients was found [20].

Very little is known about the association of infections with suicide attempts. Only few infectious agents have been studied in relation with suicide attempts including influenza B [21] and* Toxoplasma gondii* infections [22, 23]. Several studies have shown that* Toxocara* affects the brain of humans [11] and rodents [24–26]. However, it is unknown whether* Toxocara* infection is associated with suicide attempts. Therefore, we performed a case-control seroprevalence study to determine whether* Toxocara* infection is associated with suicide attempts in psychiatric outpatients in Durango City, Mexico.

## 2. Materials and Methods

### 2.1. Study Population

This case-control study was performed using stored serum samples from a recent* Toxoplasma gondii* study in psychiatric patients in Durango City, Mexico [23]. Subjects (*n* = 282) enrolled in the study were psychiatric outpatients who attended two public hospitals in Durango City: the Hospital of Mental Health “Miguel Vallebueno” and the General Hospital of the Secretary of Health. Inclusion criteria for suicide attempters were the following: (1) psychiatric outpatients with history of one or more suicide attempts; (2) those aged 18 years and older; and (3) those who accepted to participate in the study. In total, 156 suicide attempters were enrolled in the study. They were 18–61 years old (mean 34.01 ± 10.25 years) and included 119 females and 37 males. Inclusion criteria for psychiatric controls were the following: (1) psychiatric outpatients without history of suicide attempts; (2) those aged 18 years and older; and (3) those who accepted to participate in the study. Gender was not a restrictive criterion for enrollment of cases and controls. The control group included 126 (75 females, 51 males) patients aged 18–69 years (mean 38.00 ± 11.59 years).

### 2.2. General Sociodemographic, Clinical, and Behavioral Characteristics of Patients

Sociodemographic, clinical, and behavioral characteristics of the psychiatric patients were obtained with a questionnaire through a face-to-face interview. Sociodemographic items were age, gender, birthplace, educational level, occupation, and socioeconomic status. Clinical items included diagnosis of current psychiatric disease and concomitant diseases, suicidal ideation, history and number of suicide attempts, time from last suicide attempt, and method of suicide attempts. This study relied on the information about suicide attempts provided by the patients. It is unclear how sensitive the face-to-face interview to detect suicide attempts used is. In addition, other clinical data including lymphadenopathy, frequent headache, impairments in memory, reflexes, hearing, and vision, and history of blood transfusion, transplant, surgery, alcohol consumption, drug abuse, or sexual history from all participants were obtained. Behavioral items were the following: contact with animals and cat excrement, traveling, type of meat consumed, consumption of raw or undercooked meat, unpasteurized milk, dried or cured meat, unwashed raw vegetables and fruits or untreated water, frequency of eating in restaurants or fast food outlets, contact with soil, and type of flooring at home.

### 2.3. Detection of Anti-*Toxocara* Antibody

Sera of patients were kept frozen at −20°C until analyzed. All serum samples were analyzed for anti-*Toxocara* IgG antibodies with a commercially available enzyme immunoassay (EIA) “*Toxocara*” kit (Diagnostic Automation, Inc., Calabasas, CA, USA). All EIA were performed according to instructions of the manufacturer. An absorbance reading ≥ 0.3 optical density units was used as a cut-off for seropositivity. Positive and negative controls were included in each EIA. Serum samples of cases and controls were analyzed in the same run. Laboratory personnel were not blinded to study samples.

### 2.4. Statistical Analysis

We performed the statistical analysis with the software Epi Info version 7 and SPSS 15.0 (SPSS Inc., Chicago, Illinois, USA). For calculation of the sample size, we used a 95% confidence level, a power of 80%, a reference seroprevalence of 4.7% [19] as the expected frequency of exposure in controls, and an odds ratio of 3.5. The result of the sample size calculation was 106 cases and 106 controls. These values were taken as the minimum number of participants for each group. To assess the association between* Toxocara* infection and suicide attempts and other characteristics of the patients a bivariate analysis was used. The two-tailed Fisher exact test was used to compare the frequencies among the groups. Variables with *P* values <0.10 obtained in the bivariate analysis were further analyzed with stratification by gender. Statistical significance was set at *P* value <0.05.

### 2.5. Ethical Aspects

The study was performed using only residual serum samples and questionnaires from a previous survey in psychiatric outpatients [23]. The Ethical Committees of the General Hospital and the Hospital of Mental Health in Durango City approved the previous study. The purpose and procedures of the survey were explained to all participants, and a written informed consent was obtained from all of them. The additional analysis of serum samples and questionnaires was approved by the Ethical Committee of the Instituto de Seguridad y Servicios Sociales de los Trabajadores del Estado in Durango City, Mexico.

## 3. Results

One of the 156 (0.6%) suicide attempters and 1 (0.8%) of the 126 controls were positive for anti-*Toxocara* IgG antibodies (OR = 0.80; 95% CI: 0.04–13.02; *P* = 1.00). The suicide attempter seropositive for* Toxocara* had a low anti-*Toxocara* IgG antibody level (optical density units = 0.608). Similarly, the seropositive control has a low anti-*Toxocara* IgG antibody level (optical density units = 0.839).

None of the sociodemographic characteristics including age, gender, birthplace, educational level, occupation, and socioeconomic status showed an association with* Toxocara* seropositivity (Table 1). Likewise, none of the clinical characteristics studied including psychiatric disease and concomitant diseases, number of suicide attempts, time from last suicide attempt, method of suicide attempts, lymphadenopathy, frequent headache, impairments in memory, reflexes, hearing, and vision, and history of blood transfusion, transplant, surgery, alcohol consumption, drug abuse, or sexual history showed an association with* Toxocara* seropositivity. In contrast, bivariate analysis of the behavioral characteristics of the psychiatric patients (cases and controls together) showed four variables with *P* value <0.10: consumption of meat from boar (*P* = 0.07), pigeon (*P* = 0.09), and squirrel (*P* = 0.07) and consumption of raw dried goat meat (*P* = 0.06). Other behavioral characteristics of patients including contact with animals and cat excrement, traveling, consumption of unwashed raw vegetables and fruits, unpasteurized milk or untreated water, frequency of eating in restaurants or fast food outlets, and contact with soil showed *P* values >0.10 in the bivariate analysis. Stratification by gender showed that* Toxocara* seroprevalence was significantly higher in male patients with consumption of raw dried meat (1/1: 100%) than male patients without this consumption (0/88: 0%) (*P* = 0.01).* Toxocara* seroprevalence was comparable in male patients with consumption of meat from boar, pigeon, and squirrel than male patients without these consumption acts (*P* ≥ 0.05).* Toxocara* seroprevalence was similar in female patients with consumption of meat from boar, pigeon, and squirrel and raw dried goat meat than female patients without these consumption acts.

## 4. Discussion

*Toxocara* infection is one of the five more common nematodal infections of the nervous system [27]. Migration of* Toxocara* to brain does not frequently induce a recognizable neurological syndrome [16].* Toxocara* infection was associated with depression in a 65-year-old woman confirmed with psychometric tests [15]. Brain involvement during* Toxocara* infection may lead to disease and possibly to changes in behavior. Therefore, the present study aimed to determine whether* Toxocara* exposure was associated with suicide attempts in psychiatric patients. We found a low prevalence of* Toxocara* exposure among psychiatric outpatients, and* Toxocara* seropositivity was not associated with suicide attempts. In a previous study in psychiatric patients, a 4.7% seroprevalence of* Toxocara* exposure was found [19]. The lower prevalence found in the present study than that previously reported in psychiatric patients can be explained by differences in the characteristics of the patients; that is, we studied outpatients, whereas in the previous study only inpatients were examined [19]. Results of the present study suggest that* Toxocara* exposure did not represent a risk for suicide attempts in the psychiatric patients studied. However, this is the first study of its kind and results should be confirmed. Other population groups including inpatients and people living in high seroprevalence places (rural areas, tropical countries) with larger sample sizes should be studied.

We searched for contributing factors of* Toxocara* exposure in the psychiatric patients studied. We found that consumption of raw dried goat meat was associated with* Toxocara* exposure. This behavioral characteristic was the only variable associated with* Toxocara* exposure. However, the very low seroprevalence of* Toxocara* infection found among psychiatric patients did not allow us to obtain further statistically significant associations. Remarkably, consumption of goat meat was previously associated with* Toxocara* exposure in psychiatric inpatients in Durango City [19]. The fact that consumption of goat meat was associated with* Toxocara* exposure in two independent studies points towards the importance of this factor for the transmission of* Toxocara* infection to humans. In the present study, we examined new cases and a larger sample size (*n* = 282) of psychiatric patients than those (*n* = 128) included in the previous study [19]. In the present study, an association of* Toxocara* exposure with the consumption of raw dried meat from goat was found. To the best of our knowledge, this is the first report of an association of consumption of raw “dried” goat meat with* Toxocara* exposure. In a recent study, a clinical case of a 51-year-old man with lower motor neuron disease was linked to consumption of raw goat meat [28].* Toxocara* infections in goats have been poorly studied. We found only one seroprevalence report in goats. A 10.1% seroprevalence of anti-*Toxocara* antibodies was found in goats in Thessaly, Greece [29]. Further studies about* Toxocara* infection in goats are needed.

## 5. Conclusions

Results suggest that* Toxocara* exposure is not associated with suicide attempts in psychiatric outpatients in Durango City, Mexico. However, further studies with larger samples sizes to confirm our results should be conducted. The association between* Toxocara* seropositivity and consumption of raw dried goat meat deserves further investigation. Too few patients were seropositive to assess further associations between* Toxocara* exposure and sociodemographic, clinical, and behavioral characteristics of patients.

## Supplementary Material

The questionnaire used in this study is included as supplementary material.

## Figures and Tables

**Table 1:** Bivariate analysis of* Toxocara* seropositivity and a selection of sociodemographic, clinical, and behavioral characteristics of the patients studied.

Characteristic	Number of patients studied	Prevalence of *Toxocara* infection		*P* value
		Number	%	
Age groups (years)				
30 or less	100	2	2.0	0.33
31–50	151	0	0.0	
>50	31	0	0.0	
Gender				
Male	88	1	1.1	0.52
Female	194	1	0.5	
Birthplace				
Durango state	249	2	0.8	1.00
Other Mexican states	32	0	0.0	
Abroad	1	0	0.0	
Educational level				
No education	4	0	0.0	0.67
1 to 6 years	64	1	1.6	
7 to 12 years	160	1	0.6	
>12 years	54	0	0.0	
Frequent headache				
Yes	183	2	1.1	0.54
No	99	0	0.0	
Vision impairment				
Yes	159	2	1.3	0.5
No	123	0	0.0	
Cleaning cat excrement				
Yes	79	1	1.3	0.48
No	203	1	0.5	
Boar meat consumption				
Yes	11	1	9.1	0.07
No	270	1	0.4	
Pigeon meat consumption				
Yes	14	1	7.1	0.09
No	268	1	0.4	
Squirrel meat consumption				
Yes	11	1	9.1	0.07
No	271	1	0.4	
Consumption of dried goat meat				
Yes	9	1	11.1	0.06
No	273	1	0.4	
Contact with soil				
Yes	117	1	0.9	1.00
No	165	1	0.6	
Type of flooring at home				
Ceramic or wood	64	2	3.1	0.10
Concrete	210	0	0.0	
Soil	8	0	0.0	
