# Supplementary material for: Lack of Association between Toxocara Exposure and Suicide Attempts in Psychiatric Patients
Source: J Parasitol Res. 2015 Sep 21;2015:608604. doi: 10.1155/2015/608604 (PMC4592907; doi:10.1155/2015/608604)
Supplement: Supplementary file 1 — The questionnaire used in this study is included as supplementary material. [file 608604.f1.pdf]

Questionnaire-Suicide attempts

Date: Number:

Name:

Sociodemographic data:

1. Age:

2. Sex: (1) Female (2) Male

3. Birthplace:
   (1) Durango, Mexico.
   (2) Other Mexican state. Which?
   (3) Other country. Which?

4. Education
   (1) No studies.
   (2) 1-6 years.
   (3) 7-12 years.
   (4) >12 years.

5. Occupation:

(1) Agriculture<br>(2) House keeping<br>(3) Business<br>(4) Construction	(5) Employee<br>(6) Student<br>(7) Cattle raising<br>(8) None	(9) Factory worker<br>(10) Professional in health areas	(11) Professional in areas other than health<br>(12) Other. Which?

6. Socio-economic status: (1) Low. (2) Medium. (3) High.

Clinical data:

7. Psychiatric diagnosis (ICD-10):

8. Concomitant disease: (1) No (2) Yes. Which?

9. Have you ever thought to end your life? (1) Yes. (2) No.

10. Have you ever attempted to end your life? (1) Yes. (2) No.

11. If so, how many times have you attempted to end your life?

12. When was the last time?

13. How have you attempted to end your life?

14. Have you had enlarged lymph nodes in your neck or elsewhere in your body?
(1) Yes (2) No

15. Do you suffer from headaches frequently? (1) Yes (2) No

16. Do you have memory problems? (1) Yes (2) No

17. Do you have problems with your reflexes? (1) Yes (2) No

18. Do you hear well? (1) Yes (2) No

19. Do you see well? (1) Yes (2) No

20. Have you ever received any blood transfusion? (1) Yes. (2) No.

21. Have you ever received any organ transplant? (1) No. (2) Yes. Which?

22. Have you ever undergone any surgery? (1) No (2) Yes. Which?

23. Do you drink alcohol? (1) Yes (2) No

24. Do you consume drugs? (1) No (2) Yes. Which?

25. Do you have sexual promiscuity: (1) Yes (2) No.

Behavioral data:

26. Do you have cats at home? (1) Yes. (2) No.

27. Are there cats in your neighborhood? (1) Yes. (2) No.

28. Do you clean cat excrements? (1) Yes. (2) No.

29. Do you raise animals? (1) Yes. (2) No.

30. If so, which animals?

31. Have you ever traveled abroad? (1) Yes, where? (2) No.

32. Have you ever traveled in Mexico? (1) Yes, where? (2) No.

33. Do you eat pork? (1) Yes. (2) No.

34. Do you eat beef? (1) Yes. (2) No.

35. Do you eat goat meat? (1) Yes. (2) No.

36. Do you eat sheep meat? (1) Yes. (2) No.

37. Do you eat boar meat? (1) Yes. (2) No.

38. Do you eat chicken meat? (1) Yes. (2) No.

39. Do you eat turkey meat? (1) Yes. (2) No.

40. Do you eat pigeon meat? (1) Yes. (2) No.

41. Do you eat rabbit meat? (1) Yes. (2) No.

42. Do you eat venison? (1) Yes. (2) No.

43. Do you eat squirrel meat? (1) Yes. (2) No.

44. Do you eat horsemeat? (1) Yes. (2) No.

45. Do you eat opossum meat? (1) Yes. (2) No.

46. Do you eat meat from other animal? (1) Yes. Which? (2) No.

47. How cooked do you eat meat?

(1) Raw (2) Undercooked (3) Well done.

48. Have you ever eaten raw meat? (1) Yes. From which animal? (2) No.

49. Have you ever eaten raw dried meat? (1) Yes. From which animal? (2) No.

50. Do you drink unpasteurized milk? (1) Yes. From which animal? (2) No.

51. Do you eat chorizo? (1) Yes. (2) No.

52. Do you eat unwashed raw vegetables? (1) Yes. (2) No.

53. Do you eat unwashed raw fruits? (1) Yes. (2) No.

54. Do you drink untreated water? (1) Yes. (2) No.

55. How often do you eat away from home?

(1) Never. (2) 1-10 times a year. (3) More than 10 times a year.

56. Do you have contact with soil?

(1) Yes. (2) No.

57. What type of flooring do you have at home?

(1) Ceramic or wood.
(2) Concrete.
(3) Soil.
